# Al_2_O_3_ Concentration Effect on Deep Hydrodesulfurization of 4,6-Dimethyldibenzothiophene over NiWS/Al_2_O_3_–ZrO_2_ Catalysts

**DOI:** 10.1021/acsomega.4c05270

**Published:** 2024-09-16

**Authors:** Julio Cesar García-Martínez, Gerardo Chavez-Esquivel, Jesús Andrés Tavizón-Pozos, Laura Annette Romero De León, José Antonio de los Reyes Heredia

**Affiliations:** † Departamento de Biofísica, Escuela Nacional de Ciencias Biológicas, Instituto Politécnico Nacional, Prolongación de Carpio y Plan de Ayala S/N, Colonia Santo Tomás, Miguel Hidalgo, Ciudad de México 11340, México; ‡ Área Académica de Química, Departamento de Ciencias Básicas, Universidad Autónoma Metropolitana Azcapotzalco, Av. San Pablo No. 420, Nueva el Rosario, Azcapotzalco, Ciudad de México 02128, México; § Investigadores por México del CONAHCYT—Área Académica de Química, Departamento de Ciencias Básicas, Universidad Autónoma Metropolitana Azcapotzalco, Av. San Pablo No. 420, Nueva el Rosario, Azcapotzalco, Ciudad de México 02128, México; ∥ Unidad Académica de Ciencias Químicas, Universidad Autónoma de Zacatecas, Carr. Guadalajara Km. 6, Ejido La Escondida, Zacatecas 98160, México; ⊥ Departamento de Ingeniería de Procesos e Hidráulica, Universidad Autónoma Metropolitana-Iztapalapa, Av. San Rafael Atlixco No. 86, Vicentina, Iztapalapa, Ciudad de México 09340, México

## Abstract

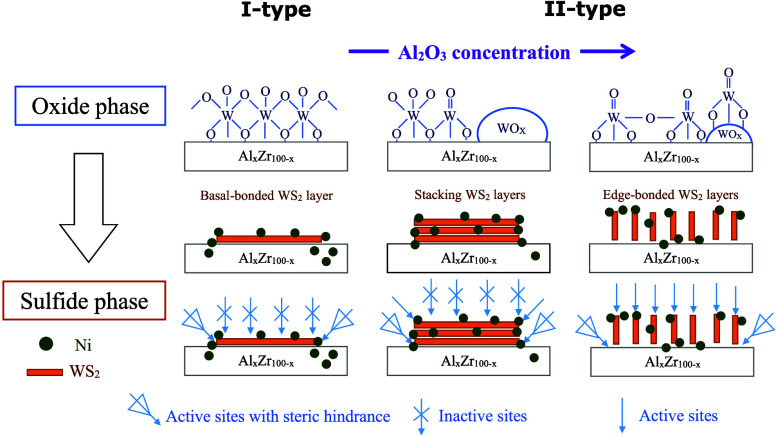

The Al_2_O_3_ concentration effect over NiWS/Al_*x*_Zr_100–*x*_ catalysts was investigated for deep hydrodesulfurization (HDS) of 4,6-dimethyldibenzothiophene (4,6-DMDBT). The sol–gel method changed the wt % Al_2_O_3_ concentrations used to synthesize the Al_*x*_Zr_100–*x*_ supports. The NiWS/Al_*x*_Zr_100–*x*_ catalysts were prepared with ammonium metatungstate hydrate and nickel(II) nitrate hexahydrate by sequential incipient impregnation, calcination, and H_2_S/H_2_ activation. The catalytic evaluation data fit a pseudo-first-order trend in the 4,6-DMDBT HDS reactions. In the oxide phase, the catalysts presented Ni and W species in tetrahedral (td) and octahedral (oh) coordination, with the oh species prevailing as a function of the Al_2_O_3_ amount. The lower amount of Al_2_O_3_ can facilitate the “Type II” NiWS phase formation by weakening the interaction of the W–O–Al bond and promoting W and Ni species sulfidation. In the sulfide phase, catalysts with (oh) coordination and surface WO*_X_* species promote the formation of WS_2_ and NiWS species during the catalyst activation step. This species favors the reaction yield, where the hydrogenation route is predominant, with the highest initial reaction rate using the NiWS/Al_25_Zr_75_ catalyst. A direct correlation was found between high hydrogenation/hydrogenolysis ratio values and low Al_2_O_3_ concentrations.

## 1 Introduction

International environmental restrictions indicate that ultralow sulfur diesel (ULSD) should reduce sulfur to 15 ppm, requiring enhanced hydrotreatment catalysts and units. Catalytic hydrodesulfurization (HDS) is utilized to eliminate sulfur from refractory molecules such as 4,6-diethyldibenzothiophene (4,6-DEDBT) and 4,6-dimethyldibenzothiophene (4,6-DMDBT) within other molecules.^1^ In HDS reactions, the functional groups (methyl) of 4,6-DMDBT generate steric hindrance that limits the direct desulfurization (DDS) pathway. Hence, 3,3-dimethylbicyclohexyl (DMBCH) formation should be enhanced by the hydrogenation (HYD) pathway. The hydrogenation capacity and functionality of NiW and NiMo catalysts on Al_2_O_3_ support have made them widely used for deep HDS treatment. Topsøe et al.^2^ have classified the catalytic active sites as NiMoS-I and NiMoS-II structures. For Ni–Mo–S systems, gallium doping improved the strength and electron-accepting properties, aligning the electronic and binding properties of the edge active sites with the corner active sites and reducing the activation energy of the 4,6-DMDBT DDS reaction.^3^ The MoS_2_ slabs are decorated with Ni, producing coordinatively unsaturated sites (CUS) ubicated at the slab edges. It is worth mentioning that Mo and W can be considered structural homologues; these metals have properties similar to those used by catalytic systems in HDS processes. In this sense, the homologous NiWS active phase can be produced in NiW/Al_2_O_3_ catalytic systems and can control the catalytic activity in HDS reactions. The support plays a fundamental role in catalytic systems utilized in hydrotreatment (HDT) processes, as it determines the textural properties and influences the arrangement, localization, and generation of surface-active species. In this sense, the active phase formation is limited by WS_2_ decoration with nickel in the lamellae edges as a function of the metal–support interactions between W or Ni species and Al_2_O_3_.^4^ Consequently, the introduction of new supports with improved physicochemical properties allows the contact force between the catalytic surface and the support to be modulated. The support modifications in catalysts used in the HDS of DBT or 4,6-DMDBT can modulate the acidity, promote the metal–support interaction, generate the active phase dispersion, and expand the pore channels, facilitating accessibility and mass transfer of the hindered dibenzothiophenes.^5,6^ Over the years, alternative synthesizing methods of catalytic materials have changed the support nature and increased the refractory molecule’s remotion. It has been demonstrated that ZrO_2_ enhances the NiMoS phase dispersion and reducibility in NiMo/Al_2_O_3_–ZrO_2_ catalytic systems. In similar systems, modification of Al_2_O_3_ weakens the Mo-support interaction and decreases acidity (Lewis), increasing the content of reducible Mo species and facilitating the formation of the NiMoS (Type II) phase.^7^ Also, the additive introduction significantly enhances reducibility and hydrogen production, with hydrogen atoms from dissociated molecules on the additive surface. This increases the number and strength of Lewis–Brønsted acid sites, thereby increasing DDS and isomerization pathways.^8,9^ The active phase promotion in this catalyst is related to the ZrO_2_ incorporation, which modifies the electron donation in the Al_2_O_3_–ZrO_2_ support by the tetragonal structure of zirconium.^10^ In the thiophene HDS reaction, using NiMo/Al_2_O_3_–ZrO_2_ catalysts changed the ZrO_2_ contents synthesized by the sonochemical (SC) and wet impregnation (WI) methods.^11,12^ The catalysts synthesized by the SC method presented a homogeneous distribution with particles lower than those synthesized by the WI method. This particle size distribution is linked to the catalytic activity. In addition, the zirconium concentration caused a catalytic system with higher activity in contrast to the free ZrO_2_ catalyst. Also, NiMo/Al_2_O_3_–ZrO_2_ catalysts have tetrahedral and octahedral species, and Mo^6+^ ion species improve in octahedral species as a function of ZrO_2_ addition, causing a low interaction between Ni–Mo and Al_2_O_3_–ZrO_2_. These catalytic systems are also used in hydrodeoxygenation reactions. In a recent study, it was observed that NiMo/TiO_2_–ZrO_2_ and NiMo/Al_2_O_3_–ZrO_2_ catalytic systems are active in the coprocessing of HDS and HDO of dibenzothiophene (DBT) and phenol.^13^ This study concluded that there is competition among these molecules for the catalytic active sites, mainly affecting the hydrogenation route of DBT.

Sol–gel synthesis methods adequately produce Al_2_O_3_–ZrO_2_ mixed since it is possible to control the textural properties and structural conformations that allow the metal–support interlinkage modulation that has shown benefits to the catalytic efficiency of NiW systems.^14^ Incorporating ZrO_2_ into alumina by the sol–gel method may generate materials with a 200 m^2^/g surface area, which is acceptable for HDS processes. The Zr addition in Y zeolite improved the acidity, the degree of sulfidation, the NiWS phase formation, and the active metal-hybrid support interactions. All of these properties improved the catalytic activity, where a high isomerization yield and a greater degree of coincidence between HYD and DSD were observed.^15^ Moreover, the presence of Zr decreased the Mo^6+^ and Ni^2+^ ion interactions with the support, increasing their availability and reducing the sulfidation temperatures. Consequently, the staking and length of the WS_2_ phase could be enhanced, which results in an adequate promotion of the NiMoS phase in NiMo/Al_2_O_3_–ZrO_2_ catalysts.^16,17^ It is worth mentioning that the NiMo/Al_2_O_3_–ZrO_2_ system has already been studied, and the NiW system seems to be another interesting option, as it is well-known to provide hydrogenation functionalities. Therefore, this work contributes to this system, which has not been studied in depth, and the results will provide early data. This work investigates the effect of Al_2_O_3_ concentration on 4,6-DMDBT HDS reactions using NiW/Al_*x*_Zr_100–*x*_ catalysts. Also, the catalysts were characterized by ultraviolet–visible (UV–vis) and Raman spectroscopies, hydrogen-temperature-programmed reduction (H_2_-TPR), high-resolution transmission electron microscopy (HRTEM), and X-ray photoelectron spectroscopy (XPS).

## 2 Experimental Procedure

### 2.1 Catalyst Synthesis

Al_2_O_3_–ZrO_2_ mixed supports with different concentrations of Al_2_O_3_ (named Al_*x*_Zr_100–*x*_), where this process was carried out by hydrolysis, with the molar ratios of alcohol/precursor, H_2_O/precursor, and HNO_3_/precursor used at 65, 20, and 0.2, respectively. The alkoxide precursors were aluminum tri-sec-butoxide [Al(OCH(CH_3_)C_2_H_5_)_3_, Aldrich 99.9%] and zirconium(IV) propoxide [Zr(OCH_2_CH_2_CH_3_)_4_, Aldrich 70%], mixed in 2-propanol with constant stirring. The alcogels were aged at rest for 1 day at room temperature for subsequent drying and calcination at 500 °C for 5 h (xerogels).^18^ All supports were impregnated with a similar tungsten charge (2.8 W atoms/nm^2^) using (NH_4_)_6_H_2_W_12_O_40_·*x*H_2_O (99.9% Aldrich) as a precursor. Then, nickel was impregnated according to the Ni/(Ni + W) = 0.41 atomic relationship using Ni(NO_3_)_2_·6H_2_O (99.9%, J. T. Baker) as a precursor. The Ni/(Ni + W) = 0.41 helps to achieve good dispersion of the active metal phases on the surface of the support. This dispersion is crucial to the accessibility of active sites, which enhances activity in HDS reactions.^19^ The wet materials are dried at 150 °C for 1 day and calcined at 350 °C for 3 h. Finally, the materials were sulfurized (activation) ex situ in a continuous bed tubular reactor under a 6 mL/min H_2_S/H_2_ (10 wt % H_2_S) flow mixture.

### 2.2 Catalyst Characterizations

The NiW/Al_2_O_3_–ZrO_2_ catalysts were characterized by UV–vis spectroscopy in a PerkinElmer Lambda 35 spectrometer provided with a diffuse reflectance integration sphere recording at 200–400 and 200–1000 nm wavelength with a 120 nm/min scan speed. The Raman spectra were made in Thermo Scientific DXR Raman Microscope equipment with a 50× objective and a 3–5 mW He–Ne laser at 532 nm. The spectra were obtained by taking the average of three points in the sample. TPR experiments were conducted in a U-shaped quartz reactor using an Altamira AMI-80 system with a thermal conductivity detector. The calcined materials were pretreated at 250 °C for 1 h under 35 mL/min helium flow. Then, the sample was cooled and heated from 30 to 900 °C with a 10 °C/min heating rate under 50 mL/min H_2_/Ar (10%, v/v) flow. The high-resolution transmission electron microscopy (HRTEM) micrographs for each catalyst were taken on JEM2010 FEG equipment with a point-by-point resolution of 0.25 nm. Before analysis, the finely ground fresh sulfide catalysts were ultrasonically dispersed on a copper grid with ethanol. The mean number of slabs and length were determined by examining ca. 500 particles extending at least ten micrographs per catalyst using previous methodology.^20^

To avoid oxidation, all catalysts were sulfidized using the same activation conditions as above and immersed in dodecane until they were analyzed in the sulfide phase. The XPS equipment and operating conditions are described in previous work.^21^

### 2.3 Catalytic Evaluation

The catalytic activity of the NiWS/Al_*x*_Zr_100–*x*_ catalysts in the 4,6-DMDBT HDS was performed in a batch stainless steel reactor (Parr 4842), for which a model mixture composed of dodecane and 300 ppm of sulfur (4,6-DMDBT) was used. The reaction conditions used were 5.52 MPa of H_2_ and 320 °C. The catalytic activity was expressed through the initial reaction rate, which was expressed in mol_4,6-DMDBT_/g_cat_·s. Before the reactions, the catalysts were sulfidized ex situ in a continuous fixed-bed reactor using a 10 mol % H_2_S/H_2_ (60 cm^3^/min) flow rate at a constant temperature of 400 °C.

## 3 Results

### 3.1 Oxide Phase Characterization

**Figure 1:**
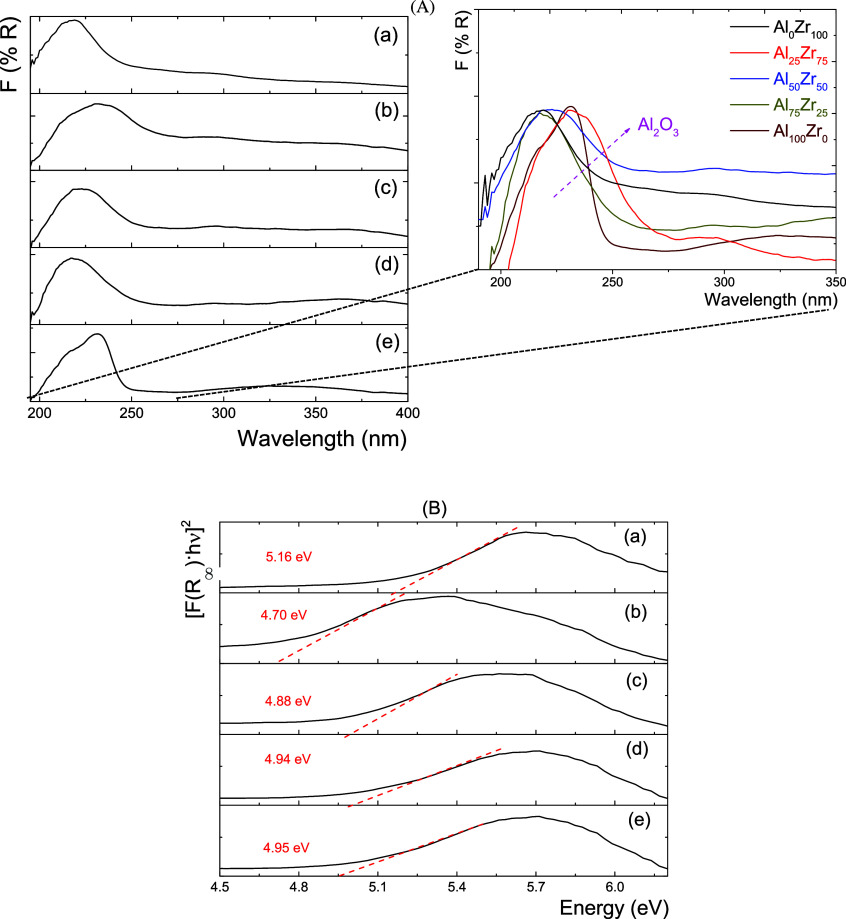
(A) UV–vis spectra of the Al_*x*_Zr_100–*x*_ support and (B) *E*_b_ values calculated by the Kubelka–Munk equation for the (a) Al_0_Zr_100_, (b) Al_25_Zr_75_, (c) Al_50_Zr_50_, (d) Al_75_Z_25_, and (e) Al_100_Zr_0_ supports.

Figure 1A(a) shows the UV–vis spectra for Al_0_Zr_100_. This support showed two bands at 232 and 338 nm, related to an O^2–^ to Zr^4+^ transition.^22^ In general, Al_25_Zr_75_, Al_50_Zr_50,_ and Al_75_Zr_25_ spectra (Figure 1A(b–d)) show two absorption bands, the first between 200 and 275 nm and the second between 275 and 425 nm. As ZrO_2_ increased in Al_*x*_Zr_100–*x*_ supports, they exhibited a shift to lower energy due to the electron excitation from the O 2p to the Zr 4d. Finally, Al_100_Zr_0_ support (Figure 1A(e)) presents a moderate absorption near 225 nm in concordance with the insulating character of the γ-Al_2_O_3_ phase; this absorption band corresponds to the metal-to-ligand charge transfer O^2–^ → Al^3+^ transition.^23^

Figure 1B presents the Al_*x*_Zr_100–*x*_ absorption edge energies (*E*_b_) obtained by the Kubelka–Munk function. The *E*_b_ values obtained were 5.16, 4.70, 4.88, 4.94, and 4.95 eV for Al_0_Zr_100_, Al_25_Zr_75_, Al_50_Zr_50_, Al_75_Zr_25_, and Al_100_Zr_0_ supports, respectively. Hence, the decrease in ZrO_2_ provoked changes in the band gap, with values near 5.0 eV, similar to previous works.^24,25^ This behavior was linked with the electron transition capacity between bands, possibly caused by a structural imperfection in the Al_*x*_Zr_100–*x*_ system by Al_2_O_3_ incorporation.^26^

**Figure 2:**
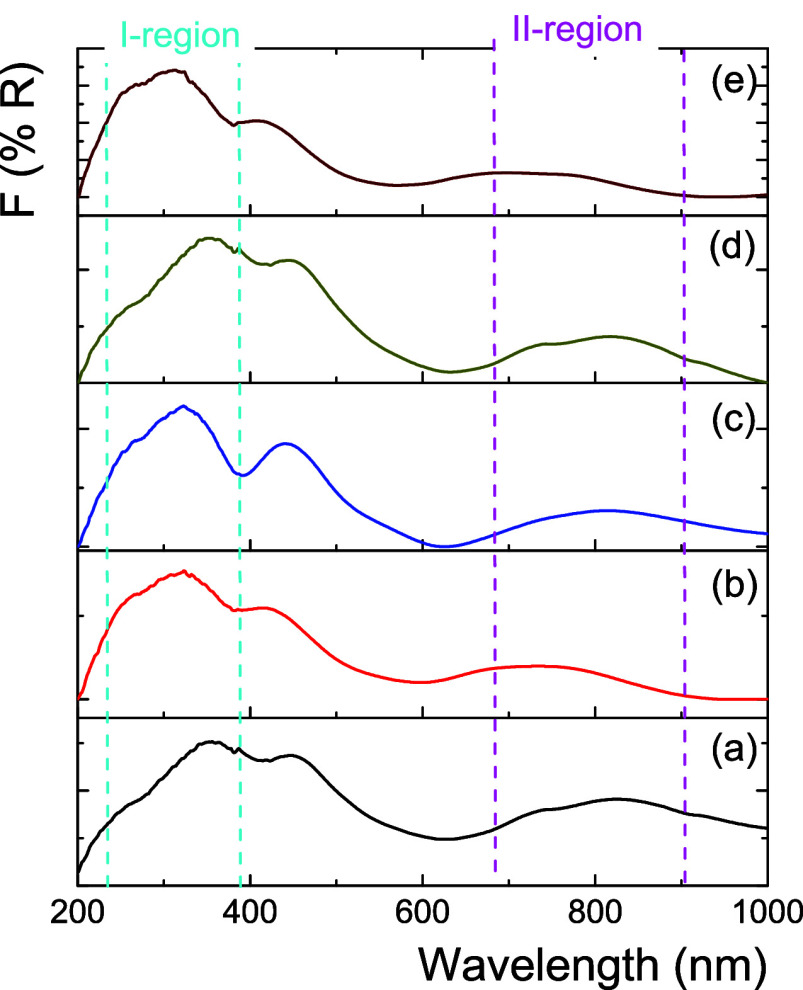
UV–vis spectra for the NiW/Al_*x*_Zr_100–*x*_ catalysts (a) NiW/Al_0_Zr_100_, (b) NiW/Al_25_Zr_75_, (c) NiW/Al_50_Zr_50_, (d) NiW/Al_75_Zr_25_, and (e) NiW/Al_100_Zr_0_ catalysts.

For the NiW/Al_*x*_Zr_100–*x*_ system, all UV–vis spectra (Figure 2) had difficulty identifying the W bands because they overlapped with the Zr band. The first corresponded to the O^2–^ → Zr^4+^ and the O^2–^ → W^6+^ charge transfer bands, whereas the second was related to the nickel d–d transitions. It was possible to observe a shift to a high wavelength with the presence of W. The *E*_b_ energy changes are due to the metal-to-ligand charge transfer O_2p_ → W_5d_–O_2p_ transition, where the oxidation temperature and WO*_X_* concentration determine the energy of this transition.^27^ Considering this, the spectra showed two peaks corresponding to tungsten oxide tetrahedral (WO*_X_*(td)) species near 280 nm and octahedral species (WO*_X_*(oh)) near 400 nm. As Zr concentration increased, the WO*_X_*(oh) species also did so due to the formation of polytungstates. In this case, it was found that for low Al_2_O_3_ concentrations (<50 wt % Al_2_O_3_), the tungsten oxide species bands would be mainly (oh) coordination. The numerous W–O–Al and W–O–Zr linkages between each WO_6_(oh) stabilize these segregated WO_*X*_ species.

Additionally, nickel incorporation changes the dispersion of W species. For example, the UV–vis spectrum of the W/Al_2_O_3_ system has shown a similar number of WO*_X_*(oh) and WO*_X_*(td) conformations.^28^ The metal-to-ligand charge transfer O^2–^ → W^6+^ transition was observed to shift to lower energy for NiW/Al_*x*_Zr_100–*x*_ catalysts with high Al_2_O_3_ amounts (>50 wt % Al_2_O_3_). This behavior is correlated to tungsten oxide species with W=O link types, which are associated with WO*_X_*(oh) species. The W–O–Al and W–O–Zr links between each WO_6_(oh) stabilize the WO*_X_* species. However, for low Al_2_O_3_ concentrations in NiW/Al_*x*_Zr_100–*x*_ catalysts, heteropolytungstates take place as a result of the WO*_X_* polytungstate becoming larger as a result of the WO_3_ cluster formation, which are small particles.

Furthermore, all NiW/Al_*x*_Zr_100–*x*_ catalyst spectra showed one absorption band with a maximum local ca. 700 nm, associated with the nickel ions with octahedral coordination (Ni^2+^(oh)), where the ^3^A_2g_ → ^3^T_1g_(F) (730 and 655 nm) and ^3^A_2g_ → ^3^T_1g_(P) (390–400 nm) transitions are the most common.^29,30^ In this sense, the NiW/Al_50_Zr_50_ (Figure 2c) and NiW/Al_75_Zr_25_ (Figure 2d) catalysts showed a redshift related to different d–d optical transitions with Ni^2+^(oh) species abundance. This Ni species coordination in the NiW/Al_*x*_Zr_100–*x*_ catalysts is related to the type of nickel segregated on the surfaces of the supports; when the catalysts are sulfided, the available Ni can decorate the WS_2_ slabs and promote the active phase. However, at high Al_2_O_3_ concentrations, it was found that nickel oxide with tetrahedral coordination (Ni^2+^(td)) is related to spinel formation, which is frequently seen in the number of sites and lattices in Al_2_O_3_-based support systems.^31^ The Zr in the Al_2_O_3_–ZrO_2_ supports may inhibit Ni incorporation into the support matrix, limiting Ni–Al_2_O_4_ spinel formation. Therefore, to produce a stable Al_2_O_3_–ZrO_2_ sesquioxide system, the cations must have similar ionic radii and coordination in the system interstices. The spectroscopy results showed octahedral coordination in the sublattice Al_2_O_3_–ZrO_2_ supports. In this sense, the ionic radii in the octahedrally coordinated Al^3+^, Zr^4+^, and Ni^2+^ species are 0.53, 0.72, and 0.69 Å, respectively.^32^ The ratios of the Al^3+^/Zr^4+^ and Al^3+^/Ni^2+^ ionic radii are 0.74 and 0.77, respectively. High ionic radii ratios caused the crystal structure to be distorted. In this sense, the Al_2_O_3_/ZrO_2_ concentration can occupy the interstices and lattices with low energy of the Al_*x*_Zr_100–*x*_ support, triggering a low affinity between the surface Al^3+^ vacancies and Ni^2+^ at the mixed support. This allows Ni particles to decorate the WS_2_ crystallite edges in future stages.

**Figure 3:**
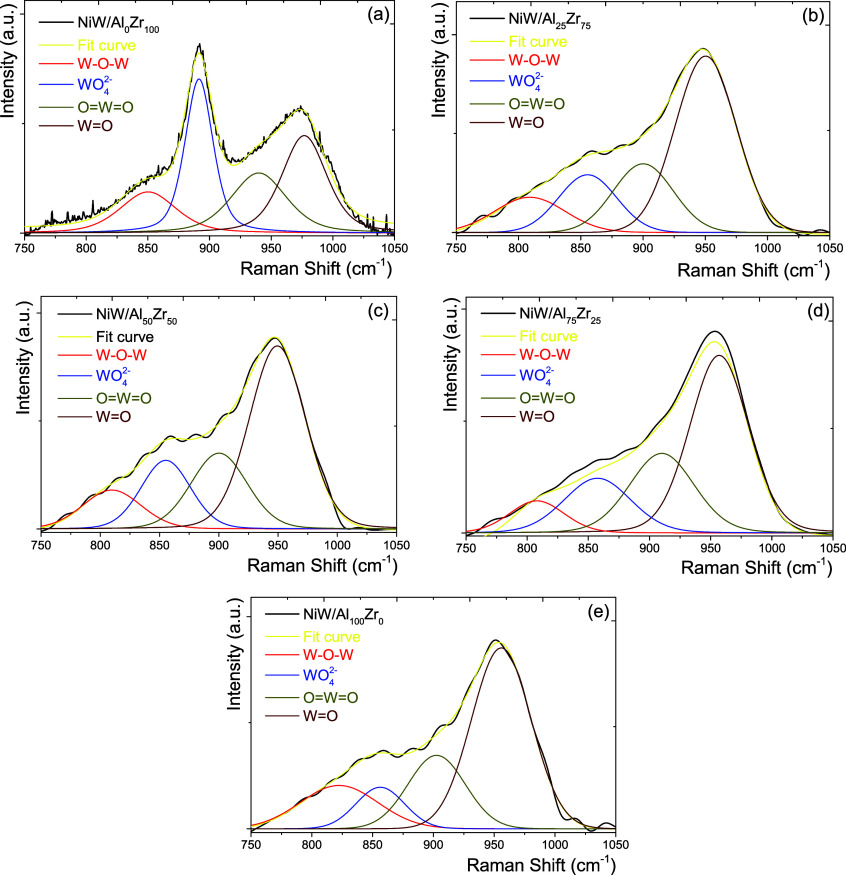
Raman spectra of catalysts calcined at 400 °C of (a) NiW/Al_0_Zr_100_, (b) NiW/Al_25_Zr_75_, (c) NiW/Al_50_Zr_50_, (d) NiW/Al_75_Zr_25_, and (e) NiW/Al_100_Zr_0_ catalysts.

Figure 3 shows the Raman spectra for NiW/Al_*x*_Zr_100–*x*_ in the oxide phase. All Raman spectra presented a wide band at 600–1100 cm^–1^ concerning WO*_X_* species, and four bands can be adjusted in this Raman broadband. At 955 cm^–1^, an active vibration band (ν_s_ [W=O]) and a wide active band at 805 cm^–1^ (ν_as_ [W–O–W]) were observed.^33^ First, monotungstate conformations showed dioxo (O=W=O) species with a WO_4_. In contrast, the octahedral coordination of polytungstates has monoxide tungsten (W=O) species between 890 and 950 cm^–1^. Additionally, the WO_3_ (monoclinic) in Raman resonance shows two bands at 720 and 805 cm^–1^ associated with W=O stretching modes.^34^ However, asymmetric W–O–W bond stretching occurs in WO*_X_* with distorted tetrahedral coordination and low polymerization levels, such as in a dimeric form.

For NiW/Al_*x*_Zr_100–*x*_ catalysts, the Raman band at 850 cm^–1^ is associated with WO_4_ decreasing with the high Al_2_O_3_ concentration. At 950–970 cm^–1^, this band can also be associated with monotungstate species with tetrahedral coordination since the center of this Raman band is too low for a stretching mode.^35^ In this sense, the superficial bonds (O=W=O or W=O) and internal bonds (W–O–W) in the NiW/Al_*x*_Zr_100–*x*_ catalysts could provide information on the WO*_X_* species dispersion as a function of the Al_2_O_3_ content. Table 1 presents the superficial/internal WO*_X_* species ratio for the NiW/Al_*x*_Zr_100–*x*_ catalysts, depending on the Al_2_O concentration. The highest intensity ratio obtained was for the NiW/Al_75_Zr_25_ catalyst, which was 2.5-fold higher than that obtained for the NiW/Al_0_Zr_100_ catalyst. This means that the W=O species and the ratio of W–O–W internal bonds decrease as a function of the Al_2_O_3_ concentration. In general, the Al_2_O_3_ concentration in the NiW/Al_*x*_Zr_100–*x*_ catalysts modulates the coexistence of superficial WO*_X_*(oh) and WO*_X_*(td) species. At high Al_2_O_3_ concentrations in the catalysts, highly distorted O=W=O and W=O from WO*_X_*(td) species were preferred on the catalyst surface.

**Table 1:** Superficial/Internal WO*_X_* Species Ratio for the NiW/Al_*x*_Zr_100–*x*_ Catalysts

catalysts	(O=W=O + W=O)/ W–O–W ratio
NiW/Al_0_Zr_100_	5.1
NiW/Al_25_Zr_75_	7.2
NiW/Al_50_Zr_50_	8.5
NiW/Al_75_/Zr_25_	11.3
NiW/Al_100_Zr_0_	6.7

**Figure 4:**
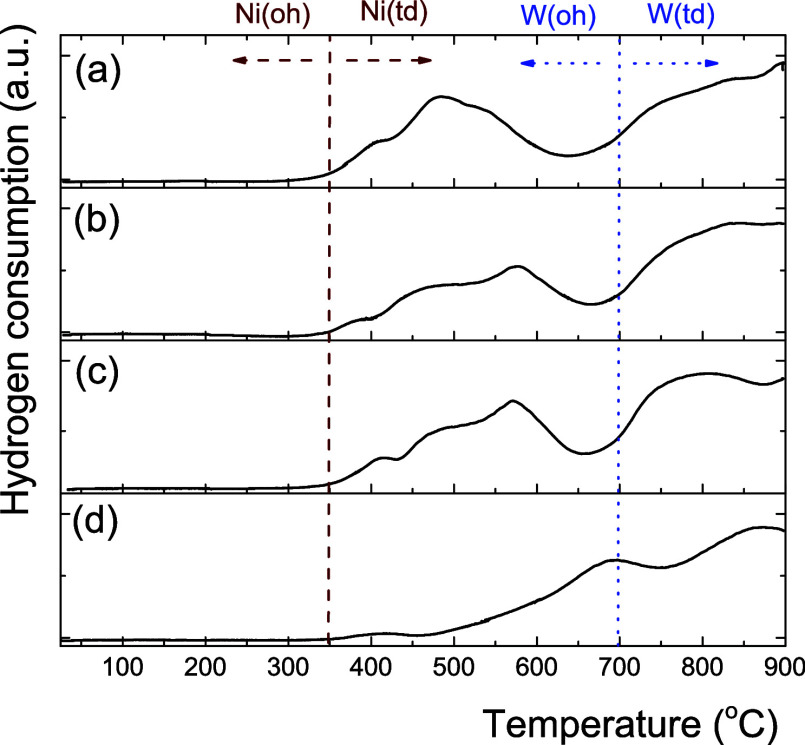
H_2_-TPR profiles for (a) NiW/Al_25_Zr_75_, (b) NiW/Al_50_Zr_50_, (c) NiW/Al_75_Zr_25_, and (d) NiW/Al_100_Zr_0_ catalysts calcined at 400 °C.

Figure 4 shows the reduction profiles of NiW/Al_*x*_Zr_100–*x*_. NiO reduction occurred from 350 to 540 °C. The Ni^2+^(oh) species require less energy to reduce than the Ni^2+^(td) species since these later strongly interact with the support.^36,37^ It has been reported that in Ni supported on Al_2_O_3_, TiO_2_, and ZrO_2_ catalysts, there is only one reduction peak with a maximum temperature in the following order: Ni/ZrO_2_ < Ni/TiO_2_ < Ni/Al_2_O_3_, indicating that Ni species have a stronger interlinkage with Al_2_O_3_.^38^ In this sense, it is possible to observe that as ZrO_2_ concentration increases, the Ni^2+^(oh) seems to increase since the reduction peaks occur near 350 °C. On the one hand, NiW/Al_100_Zr_0_ showed that the reduction of the active phase started near 500 °C due to a stronger interaction with the support than in the Zr-containing supports. For example, the NiMo/Al_2_O_3_ system prepared by calcination at 600 °C exhibits the highest ratio of penta-coordinated aluminum species; AlO_5_ can interact with W species due to proximity in the support matrix.^39^ The interaction between penta-coordinated Al and W(oh) or W(td) can influence the dispersion and stabilization of the active W species on the support surface, altering the total acidity of the catalyst.

On the other hand, the NiW/Al_75_Zr_25_ and NiW/Al_50_Zr_50_ catalysts showed two peaks, centered near 560 °C and one above 700 °C. The first one involves the reduction of NiO in octahedral and tetrahedral coordination and WO*_X_*(oh), whereas the second one mainly involves the reduction of WO*_X_*(td). The NiW/Al_25_Zr_75_ catalyst shifted to lower temperatures in its reduction signals since the first was centered at 490 °C and the second started at 650 °C. The reduction at low temperatures may be associated with the coexistence of Ni(td)–Ni(oh), polytungstates (oh), and NiWO_4_ species, all of which are responsible for the NiWS phase formation.^40^ In this sense, at low Al_2_O_3_ concentrations, the Ni–W interaction decreases, which leads to the formation of more WO*_X_*(oh) species than WO*_X_*(td) species, resulting in a reduction capacity greater than that for the NiW/Al_0_Zr_100_ catalyst.

### 3.2 Sulfide Phase Characterization

**Figure 5:**
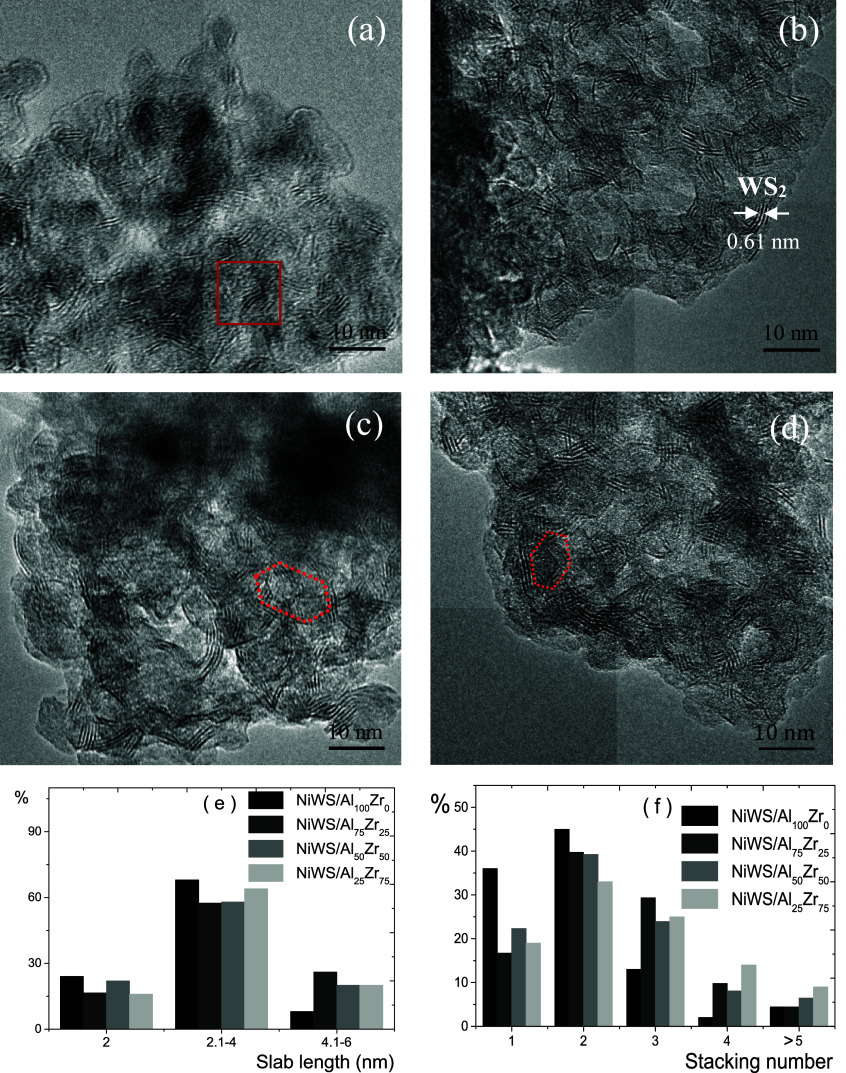
HRTEM micrographs of the (a) NiWS/Al_25_Zr_75_, (b) NiWS/Al_50_Zr_50_, (c) NiWS/Al_75_Zr_25_, (d) and NiWS/Al_100_Zr_0_ catalysts. (e) WS_2_ slab lengths and (f) stacking number of WS_2_ slabs.

Figure 5a–d shows micrographs of the NiWS/Al_*x*_Zr_100–*x*_ sulfide catalysts. The typical structuring of the slab for the WS_2_ phase could be monitored homogeneously on the surface of the Al_2_O_3_–ZrO_2_ supports with various orientations and numbers of stacks. The interplanar distance of the WS_2_ slabs was 0.61 nm with a hexagonal crystalline structure, where the W atoms are in a trigonal prismatic coordination sphere, verified with the ICDD 08–237 for the basal plane (002).^41^ For each WS_2_ crystal, the slab number and slab length are the three-dimensional stacks observed by HRTEM micrographs.

Figure 5e,f shows the WS_2_ slab length and stacking number for the NiWS/Al_*x*_Zr_100–*x*_ catalysts. All catalysts showed WS_2_ slab dispersion on Al_*x*_Zr_100–*x*_ supports with >65% frequency slabs of 2.1 to 4.0 nm in length and 2–3 stacking slabs. The WS_2_ slab size and stacking number for the NiWS/Al_*x*_Zr_100–*x*_ catalysts increased with the Zr concentration. The NiWS/Al_100_Zr_0_ catalyst presented single and double short stacking numbers. After the modification of ZrO_2_, the lengths and number of Ni-promoted WS_2_ slabs were extended, and the number of stacks was increased, although most of the stacks were double or triple.^6^ The stacking number showed a similar tendency; for the NiWS/Al_100_Zr_0_ catalyst, the main stacking number was 1 to 3. In contrast, the Zr concentration also increases the stacking number. In this regard, the NiWS/Al_25_Zr_75_ catalyst presented the highest stacking number. These results follow previous characterization techniques since Zr leads to weaker metal support interactions and WO*_X_*(oh) polytungstate formation.

**Figure 6:**
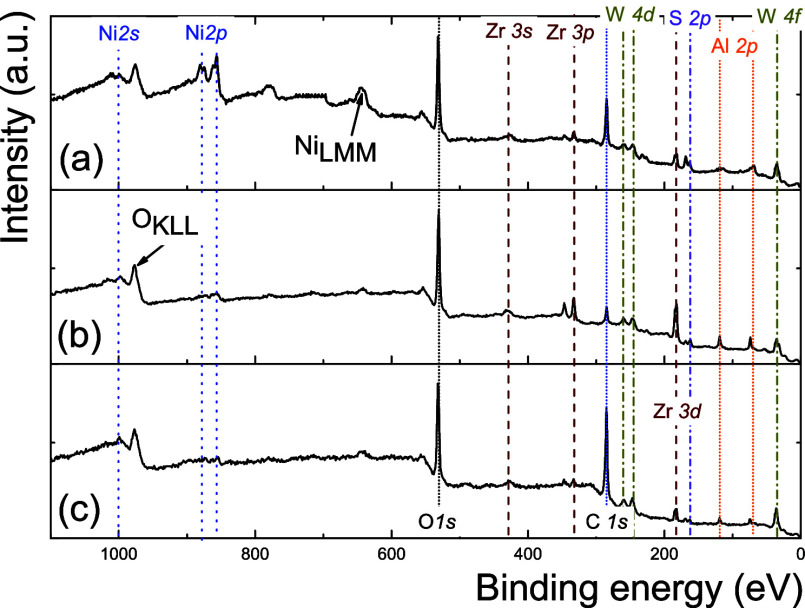
X-ray photoelectron spectroscopy (XPS) survey spectra of (a) NiWS/Al_25_Zr_75_, (b) NiWS/Al_50_Zr_50_, and (c) NiWS/Al_75_Zr_25_ catalysts.

Figure 6 presents the XPS spectra of the NiWS/Al_*x*_Zr_100–*x*_ catalysts. All spectra displayed Auger electron emissions and high-resolution regions W 4f, Al 2p, Al 2s, S 2p, Zr 3d, W 4d, C 1s, Zr 3p, Zr 3s, O 1s, Ni_LMM_, Ni 2p, O_KLL_, and Ni 2s in the following 37, 73, 120, 165, 183, 247, 260, 284, 331, 430, 530, 642, 855, 979, and 1000 eV binding energies, respectively.

**Figure 7:**
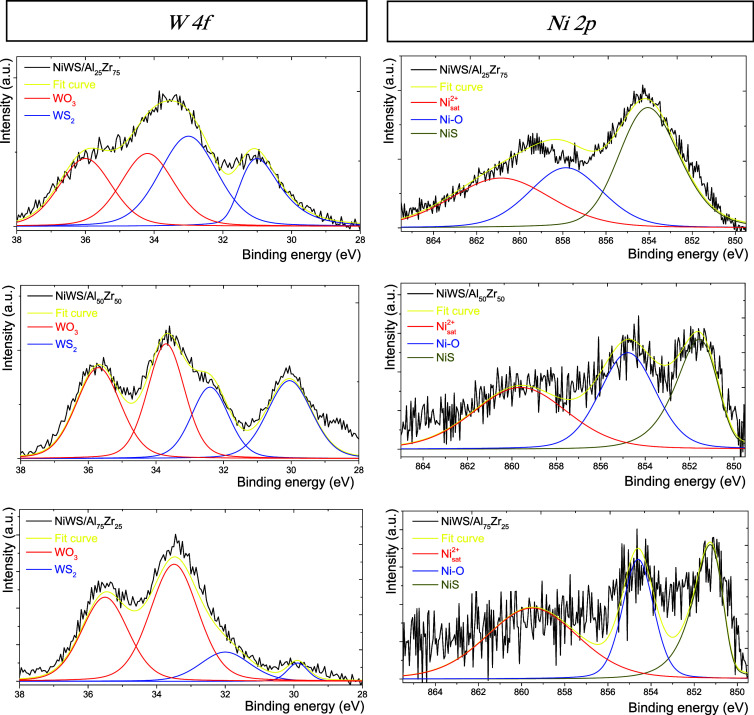
XPS spectra of W 4f and Ni 2p core level binding energies with fit curves for the NiWS/Al_25_Zr_75_, NiWS/Al_50_Zr_50_, and NiWS/Al_75_Zr_25_ catalysts.

Figure 7 shows the W 4f (right side) and Ni 2p (left side) regions for the NiWS/Al_25_Zr_75_, NiWS/Al_50_Zr_50_, and NiWS/Al_75_Zr_25_ catalysts. For the first region, four partially overlapped peaks were observed in all spectra. At 31.01 and 33.00 eV, two peaks can be related to W^4+^ or WS_2_ species for the W 4f_7/2_ level, and the doublet at 34.19 and 36.01 eV is associated with W^6+^ or WO_3_ species for the W 4f_5/2_ level.^42^

Table 2 summarizes the superficial compositions of W and Ni species from the XPS spectra. The NiWS/Al_25_Zr_75_ catalyst presented 51.9 and 48.1% W^4+^ and W^6+^ species, respectively. Also, the NiWS/Al_50_Zr_50_ and NiWS/Al_75_Zr_25_ catalysts displayed 42.7 and 26.5% WS_2_, respectively. In particular, the NiWS/Al_25_Zr_75_ catalyst presented 90% more sulfur than the NIWS/Al_75_Zr_25_ catalyst, connected with a high sulfidation capacity and sulfur species formation on the catalyst surface.^43^

**Table 2:** Superficial Composition for the W 4f and Ni 2p Core Levels for the NiWS/Al_*x*_Zr_100–*x*_ Catalysts

	superficial composition (atomic %)					
catalysts	W^4+^	W^6+^	S	NiS	Ni–O	Ni_sat_^+2^
NiWS/Al_25_Zr_75_	51.9	48.1	5.0	38.5	31.4	30.1
NiWS/Al_50_Zr_50_	42.7	57.3	3.6	29.3	33.9	36.8
NiWS/Al_75_Zr_25_	26.5	73.5	2.6	28.9	25.9	45.2

Hence, the atomic sulfur identified on the NiWS/Al_*x*_Zr_100–*x*_ catalyst surface decreased as a function of the Al_2_O_3_ concentration since the metal support interactions increased. In general, the amount of ZrO_2_ in the NiW/Al_2_O_3_–ZrO_2_ catalysts prevents nickel from migrating into the Al_2_O_3_–ZrO_2_ support network and allows the formation of WS_2_ and NiWS.^44^ In this sense, the Ni would be more available in a high ZrO_2_-containing catalyst, interacting more with the WO*_X_* species and enhancing the promotion.^45^ Consequently, the resulting sulfur species would have more labile sites and higher catalytic activity. This is in accordance with the previously exposed results.

### 3.3 Catalytic Activity Results

**Figure 8:**
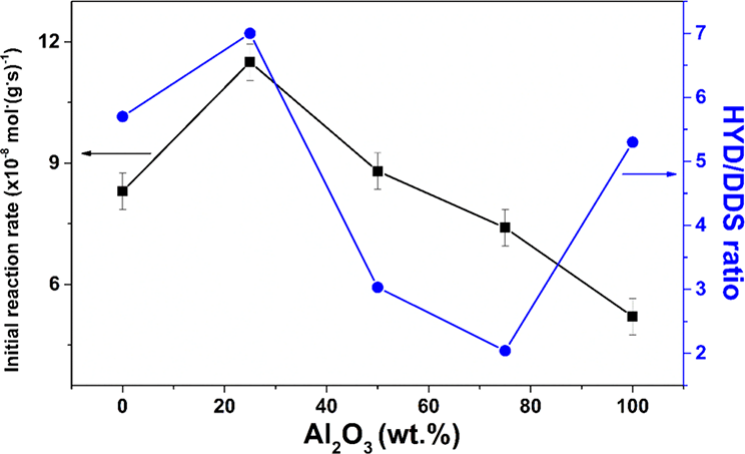
Initial reaction rate and HYD/DDS ratio in the 4,6-dimethyldibenzothiophene hydrodesulfurization for NiWS/Al_*x*_Zr_100–*x*_ catalysts as a function of the Al_2_O_3_ content.

Figure 8 presents the initial reaction rate and HYD/DDS ratio in the 4,6-DMDBT HDS using NiWS/Al_*x*_Zr_100–*x*_ catalysts with different Al_2_O_3_ concentrations. These two parameters were calculated at a 20% conversion rate for the 4,6-DMDBT HDS reaction. As the Al_2_O_3_ concentration increased, the catalytic activity decreased. In this sense, NiW/Al_25_Zr_75_ was the most active synthesized catalyst, with an initial reaction rate 2.2-, 1.5-, 1.3-, and 1.4-fold higher than those for the catalysts NiW/Al_0_Zr_100_, NiW/Al_50_Zr_50_, NiW/Al_75_Zr_25_, and NiW/Al_100_Zr_0_, respectively. Also, this material presented higher selectivity to HYD, which decreased with the alumina content but increased with NiW/Al_100_Zr_0_. The amount of NiWS phase formation and the sulfide capacity for NiWS/Al_*x*_Zr_100–*x*_ depended on the Al_2_O_3_ concentration. The Al_2_O_3_ amount decreases the NiWS phase and CUS formation and limits catalytic performance due to the nickel migration into the alumina network.^46^

The 4,6-DMDBT reaction occurred through two pathways: (i) the aromatic rings HYD route, which produced the 4,6-dimethyltetrahydrodibenzothiophene (THDBT) intermediate, which, after C–S bond incision, generated 3,3′-dimethylbicyclohexyl (DMBCH) and (ii) the DDS route, which resulted in the 3,3-bimethylbiphenyl (DMBP) formation. The acidic catalytic site not only improved the activation of 4,6-DMDBT but also facilitated the cleavage of C–C bonds of the intermediate products in the reaction.^47^ The Al_2_O_3_–ZrO_2_ system may have an improved distribution and a greater number of Brønsted acid sites compared with the individual oxides. The synergy between the two components can modify the acid–base properties of the final NiWS/Al_2_O_3_–ZrO_2_ system.

**Figure 9:**
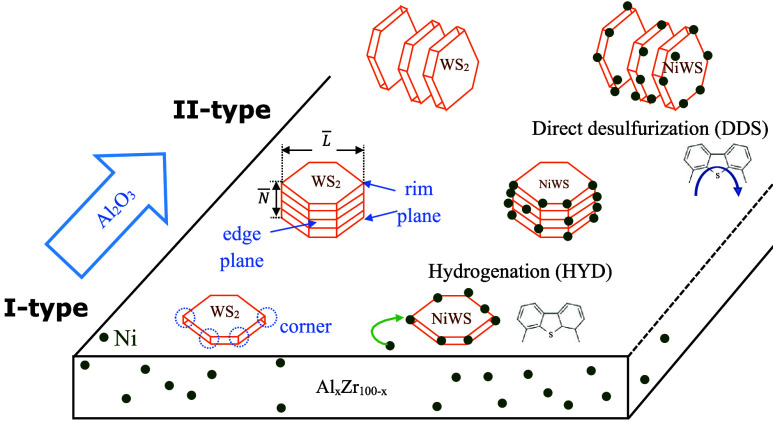
WS_2_ slabs produced on NiWS/Al_*x*_Zr_100–*x*_ catalysts with hydrogenation (HYD)-oriented and direct desulfurization (DDS) during 4,6-DMDBT HDS as a function of the Al_2_O_3_ amount.

For the NiMo/SiO_2_–Al_2_O_3_ (yolk–shell) systems, the selectivity favored the DDS route in the HDS of 4,6-DMDBT, and the performance was favored by a higher total acidity and a greater number of Brønsted acid sites on the surface of the catalyst. (NiMo/AYP-20).^48^ In this sense, as more Ni is available and the generated WO*_X_*(oh) species can be easily sulfurized on NiW/Al_25_Zr_75_, the NiWS phase was promoted, improving the HYD functionality. However, NiW/Al_100_Zr_0_ showed a higher HYD selectivity than NiW/Al_50_Zr_50_ and NiW/Al_75_Zr_25_. This would indicate that even though the active sites on NiW/Al_100_Zr_0_ are less promoted, this material still has well-dispersed labile sites that can efficiently perform the HYD reactions, as the scanning electron microscopy (SEM) results may suggest. The high dispersion of WS_2_ crystals contributes to the HYD pathway.^49^Figure 9 shows the WS_2_ stacks developed on NiWS/Al_*x*_Zr_100–*x*_ catalysts as a function of Al_2_O_3_ concentration with HYD-oriented (I-type) and DDS-oriented during 4,6-DMDBT HDS adapted from Shimada.^50^ The Topsøe model suggests that the quantity, dispersion, and stacking of the upper-lower WS_2_ stacks determine the catalytic activity and selectivity. The nature of the atoms near the WS_2_ edge determines the promoting effect, observing that the active sites differ in promoted and unpromoted catalysts.^51^ As superficially available Ni decorates the WS_2_ border sites, it produces the I-type and II-type of the active Ni–W–S phase.^52−54^ The NiWS-rich active phase generation at a catalytic site can vary as a function of the anchoring of Ni^2+^ in its molecular matrix through an electrostatic charge-balancing effect.^55^ In particular, the NiWS/Al_75_Zr_25_ catalyst showed the II-type active Ni–W–S phase preference for the DDS pathway, whereas the NiWS/Al_25_Zr_75_ I-type performed the HYD.

**Figure 10:**
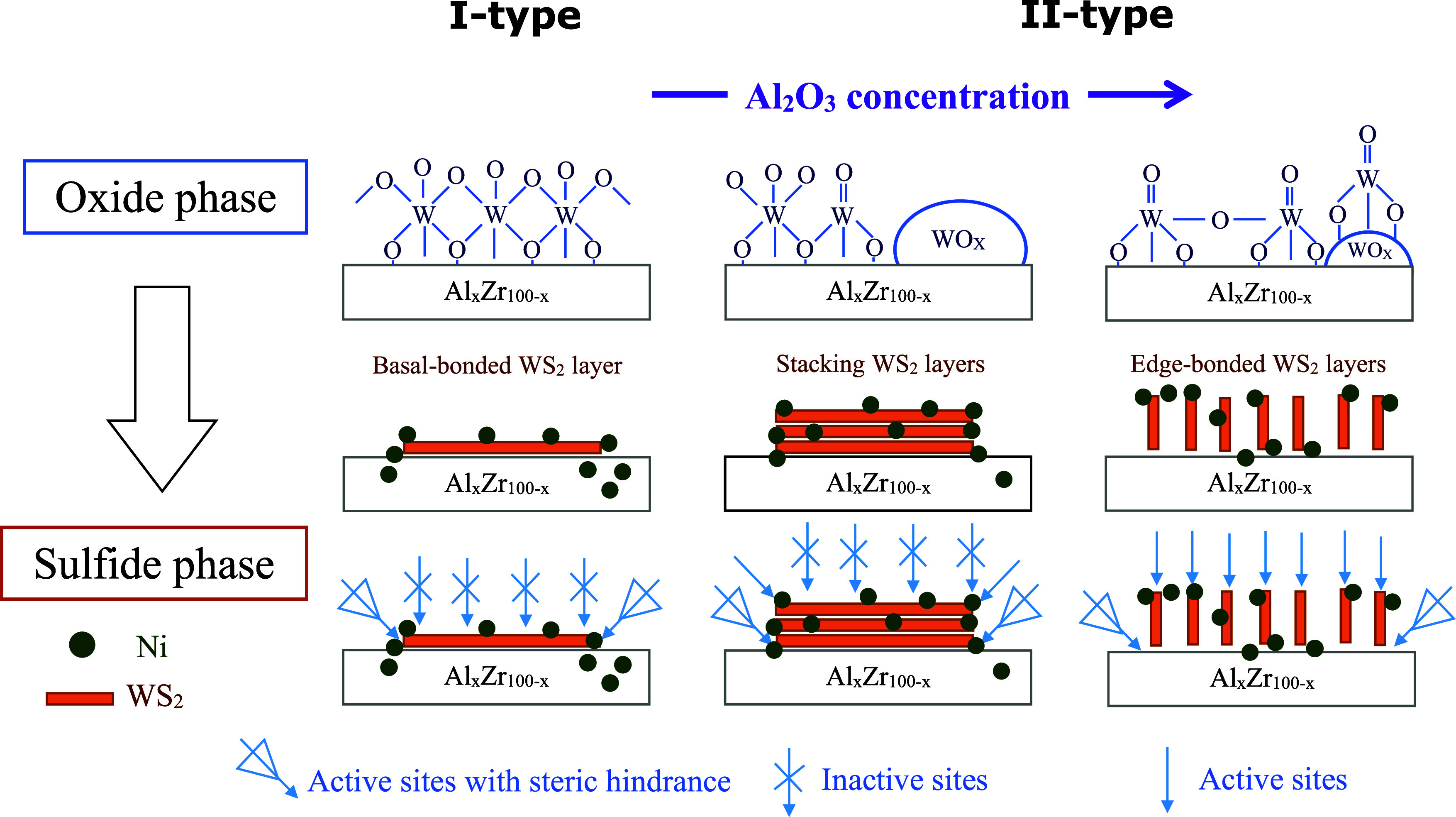
Morphologies and orientations of the WO*_X_* and WS_2_ species formed on the catalyst surface. The predominant surface oxide phase generates a particular type of sulfide phase based on Al_2_O_3_ addition in the NiWS/Al_*x*_Zr_100–*x*_ catalysts.

Figure 10 shows the WS_2_ slabs and Ni locations in the NiW/Al_2_O_3_–ZrO_2_ catalysts with various Al_2_O_3_ concentrations in the oxide and sulfide phases. Shimada^50^ and Sakashita^56^ separately proposed the I-type and II-type of the active Ni–W–S phase formation, beginning with the MoO*_X_*(WO*_X_*) surface/internal (bulk) type prevalence. In NiMo/LaAlO_*x*_ systems, the lower amount of La facilitates the formation of the II-type of the active NiMoS phase by weakening the Mo–O–Al interaction and promoting the sulfidation of the Mo and Ni species.^57^

The surface/internal type and the support crystallographic phases are connected to the I-type and II-type active phases produced during the support synthesis. In this sense, NiWS/Al_75_Zr_25_ would generate more DDS sites than the HYD sites produced by NiWS/Al_25_Zr_75_. Increasing the Al_2_O_3_ concentration in the NiW/Al_2_O_3_–ZrO_2_ catalysts generates different metal–support interactions that lead to Ni migration into the Al_2_O_3_–ZrO_2_ matrix, limiting its ability to generate HYD active phase edges. At 50 and 75 wt % Al_2_O_3_ concentrations, the NiW/Al_2_O_3_–ZrO_2_ catalysts generated species with more abundant II-type of the active Ni–W–S phase disposition compared to the NiW/Al_2_O_3_–ZrO_2_ catalysts with 0, 25, and 100 wt % Al_2_O_3_ concentrations.

## 4 Conclusions

The 4,6-DMDBT HDS reaction and the characterization results were used to monitor the Al_2_O_3_ amount effect in the NiW/Al_*x*_Zr_100–*x*_ catalysts. As the alumina concentration decreases, the metal–support interactions allow superficial polytungstate generation. Consequently, NiW supported the Al_2_O_3_–ZrO_2_ catalysts, which exhibited mainly octahedral coordination of WO*_X_* species on the oxide phase, along with more superficial Ni species. The Ni species were more available on the surface of the NiWS/Al_25_Zr_75_ material since its migration into the support was avoided or mitigated by the presence of ZrO_2_. This is due to the synergistic effect between Al_2_O_3_ and ZrO_2_, weakening the interaction between the W species and the support, increasing the content of reducible W species, and facilitating the formation of the II-type of the active Ni–W–S phase. After sulfidation, these superficial conformations allowed a synergy between the WO*_X_* species and available Ni, where the WS_2_ slab decoration with Ni was enhanced and generated the NiWS species. Additionally, catalysts with low Al_2_O_3_ concentrations promote the HYD route in 4,6-DMDBT HDS, indicating different positions and exposed active sites.
